# DDMut-PPI: predicting effects of mutations on protein–protein interactions using graph-based deep learning

**DOI:** 10.1093/nar/gkae412

**Published:** 2024-05-23

**Authors:** Yunzhuo Zhou, YooChan Myung, Carlos H M Rodrigues, David B Ascher

**Affiliations:** The Australian Centre for Ecogenomics, School of Chemistry and Molecular Biosciences, University of Queensland, St Lucia, Queensland 4072, Australia; Computational Biology and Clinical Informatics, Baker Heart and Diabetes Institute, Melbourne, Victoria 3004, Australia; The Australian Centre for Ecogenomics, School of Chemistry and Molecular Biosciences, University of Queensland, St Lucia, Queensland 4072, Australia; Computational Biology and Clinical Informatics, Baker Heart and Diabetes Institute, Melbourne, Victoria 3004, Australia; The Australian Centre for Ecogenomics, School of Chemistry and Molecular Biosciences, University of Queensland, St Lucia, Queensland 4072, Australia; The Australian Centre for Ecogenomics, School of Chemistry and Molecular Biosciences, University of Queensland, St Lucia, Queensland 4072, Australia; Computational Biology and Clinical Informatics, Baker Heart and Diabetes Institute, Melbourne, Victoria 3004, Australia

## Abstract

Protein–protein interactions (PPIs) play a vital role in cellular functions and are essential for therapeutic development and understanding diseases. However, current predictive tools often struggle to balance efficiency and precision in predicting the effects of mutations on these complex interactions. To address this, we present DDMut-PPI, a deep learning model that efficiently and accurately predicts changes in PPI binding free energy upon single and multiple point mutations. Building on the robust Siamese network architecture with graph-based signatures from our prior work, DDMut, the DDMut-PPI model was enhanced with a graph convolutional network operated on the protein interaction interface. We used residue-specific embeddings from ProtT5 protein language model as node features, and a variety of molecular interactions as edge features. By integrating evolutionary context with spatial information, this framework enables DDMut-PPI to achieve a robust Pearson correlation of up to 0.75 (root mean squared error: 1.33 kcal/mol) in our evaluations, outperforming most existing methods. Importantly, the model demonstrated consistent performance across mutations that increase or decrease binding affinity. DDMut-PPI offers a significant advancement in the field and will serve as a valuable tool for researchers probing the complexities of protein interactions. DDMut-PPI is freely available as a web server and an application programming interface at https://biosig.lab.uq.edu.au/ddmut_ppi.

## Graphical Abstract

**Graphical Abstract:**
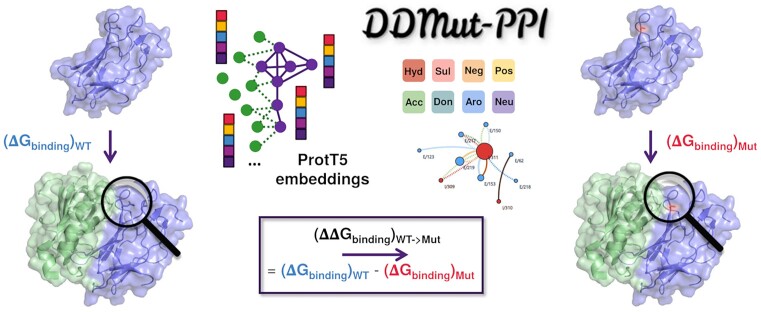

## Introduction

Protein–protein interactions (PPIs) are fundamental to many cellular processes, making them important targets for therapeutic intervention and critical focus in the study of various diseases. PPI sites contain critical areas known as hotspots, which are crucial for the strength and specificity of the interactions (1,2). The stability of these PPIs is vital for cellular equilibrium and the regulation of complex biological activities (3,4).

Mutations, particularly non-synonymous single-nucleotide polymorphisms (nsSNPs), can significantly alter these PPI interfaces. Such modifications can interfere with the typical functioning of proteins, potentially triggering a series of cellular dysfunctions and leading to diverse diseases (5). Notably, nsSNPs associated with diseases are more prevalent in PPI regions (6), highlighting the importance of these interfaces in maintaining health (5). This finding underscores the necessity for an in-depth understanding of mutation impacts on PPIs, which is crucial for the creation of targeted therapeutic strategies.

Traditional methods for exploring the effects of mutations on PPIs face challenges related to experimental complexity, cost and scalability. While computational methods provide a quicker alternative, they frequently encounter difficulties in achieving a balance between speed and precision and may not adequately represent the complex dynamics of PPI networks (7).

**Figure 1.**
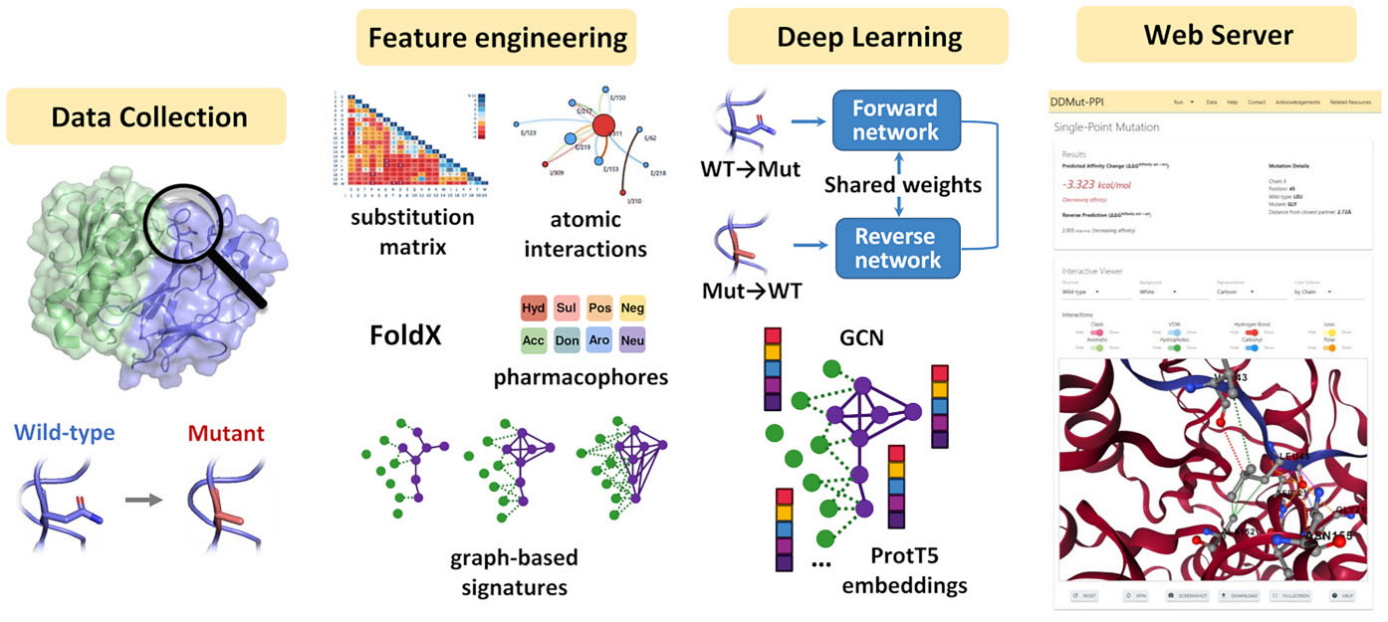
Methodology workflow overview. The methodology includes four primary steps. Initially, datasets are collected from various sources, with protein–protein complex structures sourced from the RCSB Protein Data Bank (18). Subsequently, a comprehensive set of features that captures geometric and physicochemical characteristics is derived and normalized, including the construction of the PPI interface graph. These features, together with the PPI graph, are fed into neural networks, which undergo refinement through the adjustment of hyperparameters and layers guided by training performance. This process is further validated using non-redundant blind test sets. Finally, the predictive model was deployed through an easy-to-use web interface.

In response, we have developed DDMut-PPI, a tool leveraging a graph-based deep learning approach (Figure 1) to predict the effects of single and multiple point mutations on protein binding accurately and efficiently. This model demonstrated consistent performance across mutations that strengthen or weaken interactions, offering essential insights into the molecular basis of diseases and supporting the development of novel therapeutic strategies. DDMut-PPI is freely accessible as a web server and an application programming interface (API) at https://biosig.lab.uq.edu.au/ddmut_ppi, providing a significant resource for the research community.

## Materials and methods

### Datasets

To predict the impact of single point mutations, our study utilized the S4169 dataset, derived from SKEMPI 2.0 (8), for model training to ensure comparability with existing methods. This dataset comprises 3268 mutations that reduce binding affinity (ΔΔ*G* ≤ 0 kcal/mol) and 901 mutations that enhance it (ΔΔ*G* > 0 kcal/mol), spanning 319 distinct protein complexes that are further classified into 138 types of interactions (e.g. protease–inhibitor, antibody–antigen, T-cell receptor–peptide, etc.). To address the skewed distribution of ΔΔ*G*, we incorporated hypothetical reverse mutations (9,10), where Δ*G* represents the binding free energy, as follows:


\begin{eqnarray*} {\mathrm{\Delta \Delta}}{{G}_{{\rm Mut} \to {\rm WT}}} &=& {\mathrm{\Delta }}{{G}_{{\rm WT}}} - {\mathrm{\Delta }}{{G}_{{\rm Mut}}} = - \left( {{\mathrm{\Delta }}{{G}_{{\rm Mut}}} - {\mathrm{\Delta }}{{G}_{{\rm WT}}}} \right)\nonumber\\ &=& - {\mathrm{\Delta \Delta }}{{G}_{{{\rm WT}} \to {\rm Mut}}}.\end{eqnarray*}

This adjustment expanded the original S4169 dataset by incorporating an additional 4169 hypothetical reverse mutations, each paired with its corresponding forward mutation, resulting in the S8338 dataset. This serves as the basis for training and fine-tuning our model via cross-validation. The distribution of ΔΔ*G* values is depicted in Supplementary Figure S1. For further validation, we employed several blind test sets, including the S645 dataset from the AB-Bind database (covering 645 mutations across 32 antibody–antigen complexes) (11), experimental ΔΔ*G*s for the previously unexamined MDM2–p53 complex (PDB ID: 1YCR) (12), featuring 26 interface mutations analysed through a high-throughput binding assay. Additionally, we explored deep mutational scanning datasets to evaluate the model’s predictive accuracy on novel PPIs using experimental log_2_ enrichment ratios. This encompassed the SPIKE–ACE2 dataset (13) with 418 interface mutations (PDB ID: 7KMB) and two sets from the Critical Assessment of PRedicted Interactions (CAPRI) round 26 (14,15), focusing on computationally designed inhibitors against H1N1 influenza haemagglutinin, namely T55 (1007 mutations, 285 at the interaction interface) and T56 (855 mutations, 285 at the interaction interface).

For multiple mutations, the performance of DDMut-PPI was assessed using datasets of multiple point mutations from the SKEMPI 2.0 database (8). The SM1124 dataset, comprising 900 mutations that decrease affinity and 224 that increase it, is limited to double and triple point mutations. Meanwhile, the SM595 dataset encompasses mutations that range from 4 to 27, with 442 reducing affinity and 153 enhancing it. Additionally, the SM_ZEMu dataset (16), exclusively containing mutations from SKEMPI 1.0 (17), includes 217 mutations that decrease affinity and 53 that increases it.

The wild-type structures for our analysis were sourced from the Protein Data Bank (18). Subsequently, corresponding mutant structures were generated from these wild-type structures using the MODELLER software (version 10.4) (19), employing its standard minimization pipeline. Both wild-type and mutant structures were utilized for generating the features for both forward and reverse mutations.

### Feature engineering and protein graph encoding

A comprehensive set of features was generated to capture various aspects of PPIs, considering the effects of mutations on both individual proteins and their interactions with partner proteins.

Features focusing on the individual proteins are sequence-based, derived from various substitution matrices. AAindex (20) helped in understanding how mutations alter the physicochemical and biochemical properties such as hydrophobicity, charge or size, which may further impact PPI affinity and specificity. BLOSUM and PAM (21) matrices identify conserved amino acids, where mutations might disrupt interaction sites or structural integrity essential for PPIs. Additionally, position-specific scoring matrix scores were calculated using PSI-BLAST in BLAST 2.6.0 (22) based on multiple sequence alignments, which highlights positions where mutations in highly conserved regions could affect PPI interfaces, emphasizing the importance of evolutionary conservation in PPI dynamics.

Features considering the entire protein–protein complex are structure-based. These include solvent accessibility, residue depth and secondary structure calculated using Biopython (version 1.79) (23), alongside energetic terms calculated by FoldX (24). We also analysed atomic interactions (both within the same protein and across interacting proteins) between the target residue and its neighbours using Arpeggio (25). This was complemented by assessing the mutation-induced changes in these interactions. Furthermore, we incorporated graph-based signatures from mCSM (26), utilizing a cut-off scanning algorithm (27) within a graph-theoretical representation of the local residue environment. This method helped in capturing the spatial arrangements between atom pairs, where each atom is categorized into eight pharmacophores (hydrophobics, positives, negatives, hydrogen acceptors, hydrogen donors, aromatics, sulphurs and neutrals).

To better consider the interface interactions within a 3D framework, the PPI interface was represented as a graph. Interface residues were identified as those having at least one atom within a 5.0 Å radius of atoms in the opposing protein chain. Each of these residues was represented as a graph node, enriched with ProtT5 embeddings (28) from the protein language model to account for evolutionary information. The interactions across chains at the interface were depicted as edges, generated using Arpeggio (25). Leveraging this graph representation, we computed various network analysis metrics using python-igraph (version 0.7.1) (29) to extract and interpret the complex information embedded within the PPI network. Degree and PageRank metrics were used to identify key residues; closeness and eccentricity described residue connectivity and peripherality; clustering coefficient highlighted groups of closely interacting residues; diameter and radius measured the network’s overall dimensions; Kleinberg’s authority score identified key interaction hubs; energy and entropy evaluated network stability and interaction diversity. Additionally, the distinction between central points and end points differentiated highly connected from isolated residues. Collectively, these metrics offered a comprehensive insight into the structure and dynamics of the PPI network.

### Architecture design

The DDMut-PPI model was developed using TensorFlow 2.4.2 (30). Building upon the foundational structure of the DDMut (31) Siamese network, we incorporated a graph convolutional network (GCN) within each sub-network to analyse the PPI interface graph (Supplementary Figure S2). This approach leverages ProtT5 embeddings (28), which provide a rich 1024-dimensional representation for each node, and Arpeggio's (25) detailed characterization of interactions, distinguishing 10 unique types (van der Waals, aromatic, hydrophobic, carbonyl–carbonyl, polar, ring interactions, covalent bond, hydrogen bond, halogen bond and metal complex formation) to define the edge properties. Additionally, the model employs a convolutional layer and a transformer encoder to process the graph-based signatures. In contrast, other features are processed by dense layers. These processed features are then aggregated together.

Similar to DDMut (31), an important aspect of DDMut-PPI’s architecture is the utilization of a modified contrastive loss function, inspired by Benevenuta and colleagues (32). This function is designed not only to evaluate the discrepancies between the predicted and actual ΔΔ*G* values but also to account for the anti-symmetry inherent in the relationship between forward mutations and their reverse counterparts. The loss function is defined as follows:


\begin{eqnarray*} {\rm loss} &=& \log \cosh \left( {\frac{{{\mathrm{\Delta \Delta }}{{G}_{{\rm Forward}}} - {\mathrm{\Delta \Delta }}{{G}_{{\rm Reverse}}}}}{2} - y} \right) \nonumber\\ &&+ \left| {{\mathrm{\Delta \Delta }}{{G}_{{\rm Forward}}} + {\mathrm{\Delta \Delta }}{{G}_{{\rm Reverse}}}} \right|.\end{eqnarray*}

Here, ${\mathrm{\Delta \Delta }}{{G}_{{\rm Forward}}}$ and ${\mathrm{\Delta \Delta }}{{G}_{{\rm Reverse}}}$ represent the model’s predictions for forward mutations and their reverse counterparts, respectively, while $y$ denotes the experimentally measured ΔΔ*G* for the forward mutation. Ideally, a model that perfectly captures the anti-symmetry and accuracy of these mutations would satisfy the conditions ${\mathrm{\Delta \Delta }}{{G}_{{\rm Forward}}} = y$ and ${\mathrm{\Delta \Delta }}{{G}_{{\rm Reverse}}} = - {\mathrm{\Delta \Delta }}{{G}_{{\rm Forward}}}$, resulting in a zero loss.

Throughout the training phase, the model’s hyperparameters were optimized to achieve optimal performance, guided by a leave-one-binding-site-out cross-validation strategy on the training dataset.

### Evaluation metrics

We used seven different metrics to evaluate the model performance from diverse aspects. The linear relationship between experimental and predicted ΔΔ*G* was evaluated by *R*-squared and Pearson correlation (denoted by *R*^2^ and *r*, respectively), the non-parametric relationship was evaluated by Spearman’s rank and Kendall’s rank correlation coefficient (denoted by *ρ* and *τ*, respectively), and lastly, the errors were evaluated by root mean squared error (RMSE), mean absolute error (MAE) and mean signed error (MSE).

## Web server

DDMut-PPI has been made accessible as a free and intuitive web server, hosted at https://biosig.lab.uq.edu.au/ddmut_ppi/. The frontend is built using MaterializeCSS (version 1.0.0), while the backend functionality is powered by the Flask module (version 2.0.3) from Python 3.6.13. The web server is hosted on a Linux machine running Nginx.

### Input

DDMut-PPI offers a versatile platform for predicting the impact of both single and multiple point mutations. To submit a job for prediction, users are required to provide a wild-type structure, either by uploading a PDB file or by entering a valid PDB accession code. Additionally, users have the convenience of providing an email address to receive notifications upon completion of their analysis, ensuring that they are promptly informed when results are available.

For ‘Single Mutation’ analyses (referenced in Supplementary Manual S1, Part A), mutations can be specified as a text string, such as ‘L45G’, where the format consists of the wild-type residue’s one-letter code, its position and the mutant residue’s one-letter code, along with the associated chain identifier. Alternatively, users can submit a list of up to 500 mutations either by directly inputting the text or by uploading a file. The platform also provides the option to include predictions for hypothetical reverse mutations.

For more extensive studies, the ‘Interface Analysis’ feature (detailed in Supplementary Manual S1, Part B) enables users to conduct alanine scanning or saturation mutagenesis on interface residues.

When exploring the effects of ‘Multiple Mutations’ (Supplementary Manual S2), users should delineate each mutation in the variant with a semicolon, such as ‘I D46A; I R48K’, indicating multiple mutations (D46A and R48K in this example) occurring on the same chain, I. Alternatively, users can perform a systematic evaluation of all permutations of double and triple point mutations on one side of a PPI interface.

For guidance on submitting jobs and navigating the platform, a detailed help page is accessible at https://biosig.lab.uq.edu.au/ddmut_ppi/help.

### Output

For the ‘Single Mutation’ analysis, the web interface displays the predicted ΔΔ*G* values, and details about the wild-type residue’s surroundings. It also includes an interactive 3D visualization powered by the NGL Viewer (33), which highlights inter-residue interactions (Supplementary Manual S3, Part A). Additionally, a 2D interaction graph, generated using python-igraph (version 0.7.1) (29), offers a comprehensive view where nodes symbolize interacting residues (differentiated by chain with distinct colours), and edges represent the types of interactions (with dashed lines for those present in the wild-type structure and solid lines for the mutant).

For analyses involving a ‘Mutation List’, the outcomes are compiled into a downloadable table, as shown in Supplementary Manual S3, Part B. This table includes direct links to detailed pages for each mutation, mirroring the information found in the ‘Single Mutation’ output.

The ‘Interface Analysis’ feature, detailed in Supplementary Manual S3, Part C, showcases predictive results through bar charts for alanine scanning (see Supplementary Manual S3, Part D) and heat maps for saturation mutagenesis (refer to Supplementary Manual S3, Part E), facilitating an intuitive understanding of the interaction landscape.

In the ‘Multiple Mutations’ scenario, the results are summarized as a downloadable table. This table allows for the selection of specific entries for visualization in the interactive 3D viewer, as highlighted in Supplementary Manual S4. Furthermore, the platform supports a comprehensive systematic evaluation, automatically generating all possible double and triple mutant permutations on one side of the interface. The results page presents the top 100 mutations that either increase or decrease affinity.

### API

DDMut-PPI is equipped with an API designed to streamline its incorporation into diverse research workflows. Upon submission, each job is allocated a unique identification number. This number facilitates the monitoring of job progress and the retrieval of results via the web interface. The inputs required by the API are consistent with those used on our web platform. Detailed instructions and examples employing curl and Python are available at https://biosig.lab.uq.edu.au/ddmut_ppi/api for further reference.

### Processing time

We assessed DDMut-PPI’s efficiency by comparing its processing time with that of mCSM-PPI2 (34) and DGCddG (35), specifically for single point mutation analysis, alanine scanning and saturation mutagenesis, as detailed in Supplementary Table S1.

## Validation

DDMut-PPI demonstrated its capability to accurately predict the impacts of both single and multiple point mutations (Table 1). Our assessment employed a diverse array of metrics to evaluate the linear and non-parametric relationships, as well as the discrepancies between the predicted and actual ΔΔ*G* values, ensuring a thorough analysis of the model’s predictive performance.

**Table 1.** DDMut-PPI performance under leave-one-complex-out cross-validation (CV1) and leave-one-binding-site-out cross-validation (CV2) on S8338 and blind test sets for predicting effects of single and multiple mutations

		Linear relationship metrics		Non-parametric relationship metrics		Error metrics (unit: kcal/mol)		
		*R* ^2^	Pearson	Spearman	Kendall	RMSE	MAE	MSE
Single mutation	S8338 CV1	0.56	0.75	0.75	0.58	1.33	0.87	0.007
	S8338 CV2	0.44	0.67	0.70	0.53	1.51	0.96	−0.04
	S645	0.34	0.60	0.66	0.49	1.60	0.84	0.28
	MDM2–p53	−0.17	0.37	0.37	0.27	0.64	0.52	0.12
	SPIKE–ACE2	^a^	0.37	0.45	0.31	^a^	^a^	^a^
	T55	^a^	0.33	0.30	0.20	^a^	^a^	^a^
	T56	^a^	0.31	0.41	0.27	^a^	^a^	^a^
Multiple mutations	SM1124	0.65	0.83	0.80	0.61	1.51	1.13	−0.28
	SM595	0.18	0.71	0.69	0.52	2.56	1.85	−1.45
	SM_ZEMu	0.33	0.72	0.76	0.57	2.04	1.48	−0.85

^a^Since the labels of these datasets indicate log_2_ of the enrichment ratio instead of ΔΔ*G*, *R*^2^ and error metrics were not measured.

### Predicting the effects of single point mutations

For predicting the effects of single point mutations, we tuned the hyperparameters under protein-level cross-validation on the training set S8338. Our approach incorporated two distinct cross-validation strategies to ensure robustness:

1. Leave-one-complex-out (CV1): Here, the dataset was partitioned into 319 folds, each encompassing mutations within a unique protein–protein complex identified by the same PDB ID.
2. Leave-one-binding-site-out (CV2): In this strategy, the dataset was divided into 138 folds based on distinct binding site types from the SKEMPI 2.0 database, such as antibody–antigen interactions, protease–inhibitor interactions, etc.

**Figure 2.**
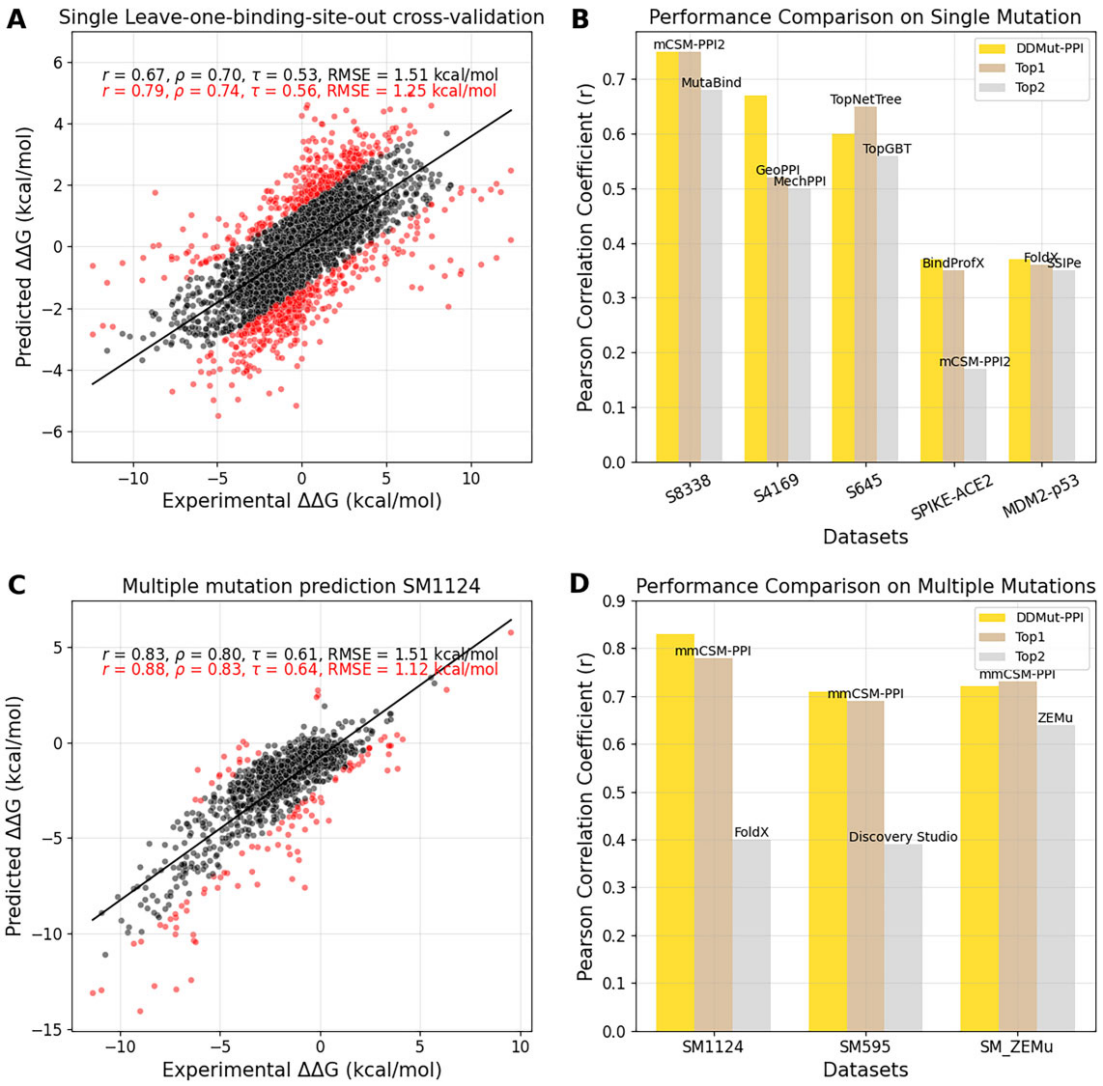
DDMut-PPI performance on predicting the effects of single and multiple mutations on PPIs. (**A**) Model performance under leave-one-binding-site-out cross-validation for the single point mutation training set. This method ensures minimal redundancy by assigning protein complexes with identical hold-out types of binding sites to the same validation fold. Outliers, constituting the furthest 10% from the best fit line, are distinctly marked in red. The correlations are denoted as *r* for Pearson, *ρ* for Spearman’s rank, and *τ* for Kendall’s rank. (**B**) Comparative analysis of DDMut-PPI against the top two methods across various single mutation benchmark datasets. The full benchmarking results are shown in Supplementary Tables S2–S5. (**C**) The performance on the multiple point mutation dataset SM1124 by aggregating predictions for individual mutations. (**D**) A comparative evaluation of DDMut-PPI with the leading two methods across benchmark datasets involving multiple point mutations. The full benchmarking results are shown in Supplementary Table S9.

Under the CV1 strategy, DDMut-PPI achieved a Pearson correlation of 0.75 (RMSE: 1.33 kcal/mol), while the CV2 approach yielded a slightly lower correlation of 0.67 (RMSE: 1.51 kcal/mol) as shown in Table 1 and Figure 2A. These results highlight the inherent challenges in achieving broader generalization across different interaction types. Nonetheless, DDMut-PPI displayed competitive performance when benchmarked against alternative methods (34–37) under similar protein-level cross-validation conditions, as detailed in Supplementary Table S2.

To compare with other methods, DDMut-PPI was then evaluated on three different blind test sets, including 645 mutations in antibody–antigen complexes (11), 26 mutations in the MDM2–p53 complex (12) and 418 mutations in the SPIKE–ACE2 complex (13). DDMut-PPI achieved competitive performance against the top methods (12,24,26,34,35,38–44) across these diverse datasets (Figure 2B and Supplementary Tables S3–S5). Additionally, DDMut-PPI was further validated on deep mutational scan datasets from CAPRI round 26 (14,15), encompassing 1007 and 855 mutations on the *de novo* influenza inhibitors T55 and T56, respectively (Supplementary Tables S6). It is noteworthy that the SPIKE–ACE2, T55 and T56 datasets rely on deep mutational scanning and differ from our ΔΔ*G*-based training data, which may contribute to lower prediction performance. Additionally, the distinct interaction patterns observed in MDM2–p53, T55 and T56, which fall into specific ECOD H-level groups (35,45,46) (totalling 3751 groups across 206 556 PDB structures as of 28 November 2023), were not represented in the SKEMPI 2.0 training dataset. This dataset is predominantly composed of protease–inhibitor, antibody–antigen and interactions of the T-cell receptor with a peptide in the major histocompatibility complex, accounting for nearly half of its composition. This poses additional challenges for prediction due to the diversity and complexity of these structural classifications.

An ablation study was conducted to ensure the significance and uniqueness of each architectural component within DDMut-PPI. Through systematic deactivation of individual sub-components followed by model retraining and evaluation, we observed a decline in overall performance (Supplementary Table S7). This pattern underscores the essential contribution of each element to the model’s overall robustness.

To further examine the feature importance, we randomly shuffled each feature in the blind test set S645. Notably, two features stood out for their pronounced effect on performance: the ΔΔ*G* calculated by FoldX (24) and the Δauthority score, with the Pearson correlation coefficient dropping from 0.343 to 0.300 and 0.330, respectively (see Supplementary Table S8). While FoldX also predicts ΔΔ*G*, Δauthority score measures the difference in Kleinberg’s authority score (47) between the wild-type and the mutant interaction network graph generated by python-igraph (version 0.7.1) (29). It assesses the changes in importance or centrality of nodes within a graph. In the context of PPI networks, this difference could evaluate the changes in importance of residues within the interaction interface, taking into account the structure of connections and the influence of neighbouring nodes, making it a critical contributor to the model’s predictive accuracy.

### Predicting the effects of multiple point mutations

To predict the effects of a multiple point mutation, DDMut-PPI employs an additive approach, aggregating the effects of individual single point mutations to predict the overall outcome. This method yielded a Pearson correlation of 0.83 (RMSE: 1.51 kcal/mol) on the SM1124 dataset, which comprises double and triple point mutations from the SKEMPI 2.0 database (Table 1 and Figure 2C). Additionally, the model demonstrated a Pearson correlation of 0.71 and an RMSE of 2.56 kcal/mol on the SM595 dataset, encompassing variants with 4–27 mutations, outperforming other methods (Figure 2D and Supplementary Table S9). The comparative decrease in performance on the SM595 dataset highlights the challenge of accurately predicting the joint effects of a higher number of mutations, which may deviate from the straightforward sum of individual mutation impacts. Moreover, DDMut-PPI displayed a more consistent performance across mutations that either decrease or increase affinity, highlighting its balanced accuracy in comparison to other models (Supplementary Table S10). We further evaluated DDMut-PPI on variants with 2–15 mutations that were derived from SKEMPI 1.0 (17), where it also exhibited competitive performance when benchmarked against mmCSM-PPI (48) and FoldX (24), both of which also employ an additive approach for their evaluations (Figure 2D and Supplementary Table S9).

## Conclusion

Here we present DDMut-PPI, a web server to predict the effects of single and multiple point mutations on PPIs. Building upon the Siamese network architecture utilizing both forward and hypothetical reverse mutations to account for model anti-symmetry, DDMut-PPI added a GCN to better capture the importance of residues at the interface based on a 2D interaction network graph. DDMut-PPI’s generalizability is constrained by the diversity of interaction types in the SKEMPI 2.0 database (8), which may not fully represent the broader range of PPIs encountered in several blind test sets. Additionally, given the current research gap in high-precision mutant modelling tools, our reliance on MODELLER (19) and its optimization energies to generate mutant structures may introduce structural errors. While MODELLER optimizes the mutant side chain using conjugate gradient and refines it with molecular dynamics, it does not account for potential structural changes in neighbouring residues. This limits the model’s ability to reflect true structural alterations, even though these structural inaccuracies might be slightly mitigated by the GCN architecture due to its robustness to noise. Despite these challenges, DDMut-PPI still shows competitive performance against other methods on non-redundant testing datasets, particularly on the effects of multiple point mutations, and more balanced performance between mutations that decrease and increase binding affinity. We believe that DDMut-PPI would be a valuable resource for researchers and clinicians looking to explore the complex dynamics of protein interactions and their implications for health and disease. DDMut-PPI is freely available as a user-friendly web server at https://biosig.lab.uq.edu.au/ddmut_ppi/.

## Supplementary Material

gkae412_Supplemental_File

## Data Availability

This website is free and open to all users and there is no login requirement. DDMut-PPI web server is available at https://biosig.lab.uq.edu.au/ddmut_ppi/.
