# Supplementary material for: DDMut-PPI: predicting effects of mutations on protein–protein interactions using graph-based deep learning
Source: Nucleic Acids Res. 2024 May 23;52(W1):W207–14. doi: 10.1093/nar/gkae412 (PMC11223791; doi:10.1093/nar/gkae412)
Supplement: gkae412_Supplemental_File [file gkae412_supplemental_file.docx]

SUPPLEMENTARY MATERIAL

# DDMut-PPI: predicting effects of mutations on protein-protein interactions using graph-based deep learning

Yunzhuo Zhou^1,2^, YooChan Myung ^1,2*^, Carlos H.M. Rodrigues^1^, David B. Ascher^1,2*^

^1^The Australian Centre for Ecogenomics, School of Chemistry and Molecular Biosciences, University of Queensland, St Lucia, Queensland 4072, Australia

^2^Computational Biology and Clinical Informatics, Baker Heart and Diabetes Institute, Melbourne, Victoria, Australia

*To whom correspondence should be addressed D.B.A. Tel: +61 90354794; Email: [d.ascher@uq.edu.au](mailto:d.ascher@uq.edu.au)

# Table S1. Comparing processing time of different tools on PDB ID 1FIN (298 amino acids on chain A; 260 amino acids on chain B)

|  | Single Mutation | | Alanine Scanning  (79 interface mutations) | Saturation Mutagenesis  (1634 interface mutations) |
| --- | --- | --- | --- | --- |
|  | H119A_A | K266A_B |  |  |
| DDMut-PPI | 67 s | 56 s | 47 min 4 s | 12 h 6 min |
| mCSM-PPI2 | 40 s | 38 s | 45 min 49 s | 10 h 25 min |
| DGCddG^*^ | 113 s | 113 s | 35 min 41 s | 11 h 49 mins |

^*^87 s for model loading, 26 s for predicting each mutation (1).

# Table S2. Comparing protein-level cross-validation performance on single point mutation training set.

|  | S4169 | | S8338 | |
| --- | --- | --- | --- | --- |
| Methods | Pearson | RMSE | Pearson | RMSE |
| DDMut-PPI CV1 | 0.67 | 1.33 | 0.75 | 1.33 |
| mCSM-PPI2 CV1^^^ | - | - | 0.75 | 1.30 |
| MutaBind CV1^#^ | - | - | 0.68 | 1.41 |
| DDMut-PPI CV2 | 0.54 | 1.50 | 0.67 | 1.51 |
| mCSM-PPI2 CV2^^^ | - | - | 0.67 | 1.39 |
| MutaBind CV2^#^ | - | - | 0.57 | 1.57 |
| TopNetTree^*^ | 0.41 | 1.60 | 0.59 | 1.65 |
| GeoPPI^*^ | 0.52 | 1.48 | 0.68 | 1.49 |
| FoldX^*^ | 0.27 | 2.73 | 0.44 | 2.73 |
| DGCddG^*^ | 0.38 | 1.63 | 0.55 | 1.68 |
| MechPPI^+^ | 0.50 | 1.58 | 0.71 | 1.49 |

^^^Results are taken from (2).

^#^Results are taken from (3).

^*^Results are taken from (1). Similar complexes classified into same group were split into the same fold, but the grouping strategy was different for each method, and was not provided in the original manuscript.

^+^Results are taken from (4).

Table S3. Performance comparison on ABbind dataset S645. Performance of other methods are obtained from (5,6).

| Methods | Pearson |
| --- | --- |
| DDMut-PPI test | 0.60 |
| DDMut-PPI test non-redundant^*^ | 0.34 |
| TopNetTree | 0.65 |
| TopGBT | 0.56 |
| mCSM-AB | 0.53 |
| TopCNN | 0.53 |
| Discovery Studio | 0.45 |
| mCSM-PPI | 0.35 |
| FoldX | 0.34 |
| STATIUM | 0.32 |
| DFIRE | 0.31 |
| bASA | 0.22 |
| dDFIRE | 0.19 |
| Rosetta | 0.16 |

^*^For all machine learning methods predicting mutation effects on PPI, S4169 was used as the training set, which also includes antibody-antigen complexes. Here for DDMut-PPI test non-redundant, we removed all antibody-antigen complexes in S4169, retrained DDMut-PPI, and evaluated on S645.

# Table S4. Performance comparison on MDM2-p53 blind test set. Performance of other methods are obtained from (2).

| Methods | Pearson | Kendall | RMSE |
| --- | --- | --- | --- |
| DDMut-PPI | 0.368 | 0.269 | 0.639 |
| mCSM-PPI2 | 0.353 | 0.231 | 0.592 |
| mCSM-PPI | 0.225 | 0.173 | 0.826 |
| BeAtMuSiC | -0.226 | -0.139 | 0.913 |
| BindProfX | 0.361 | 0.244 | 1.086 |
| FoldX | -0.140 | -0.062 | 0.903 |
| iSEE | 0.238 | 0.176 | 0.805 |

# Table S5. Performance comparison on SPIKE-ACE2 dataset. Performance of other methods are obtained from (1).

| Methods | Pearson | Kendall |
| --- | --- | --- |
| DDMut-PPI | 0.369 | 0.310 |
| mCSM-PPI2 | 0.0498 | 0.0376 |
| DGCddG | 0.153 | 0.159 |
| GeoPPI | -0.0423 | -0.032 |
| SSIPe | 0.168 | 0.102 |
| FoldX | 0.354 | 0.326 |

# Table S6. Performance comparison on CAPRI (T55 and T56) blind test sets.

|  | T55 full | | T55 interface | | T56 full | | T56 interface | |
| --- | --- | --- | --- | --- | --- | --- | --- | --- |
| Methods/Group | Kendall | Pearson | Kendall | Pearson | Kendall | Pearson | Kendall | Pearson |
| DDMut-PPI | 0.200 | 0.326 | 0.220 | 0.338 | 0.274 | 0.301 | 0.307 | 0.408 |
| mCSM-PPI2 | 0.416 | 0.548 | 0.352 | 0.451 | 0.320 | 0.402 | 0.295 | 0.378 |
| FoldX | 0.316 | 0.382 | 0.288 | 0.384 | 0.192 | 0.223 | 0.285 | 0.353 |
| SSIPe | - | - | 0.111 | - | - | - | 0.102 | - |
| BindProfX | - | - | -0.108 | - | - | - | -0.021 | - |
| DGCddG | - | - | -0.022 | - | - | - | -0.060 | - |
| mCSM-PPI | 0.16 | - | - | - | 0.13 | - | - | - |
| MutaBind | 0.41 | - | - | - | 0.30 | - | - | - |
| MMPBSA | 0.19 | - | - | - | 0.08 | - | - | - |
| Julie Mitchell* | 0.061 | - | 0.159 | - | 0.056 | - | 0.092 | - |
| Chaok Seok* | 0.080 | - | 0.142 | - | 0.035 | - | 0.133 | - |
| Martin Zacharias* | 0.098 | - | 0.216 | - | 0.029 | - | 0.132 | - |
| Paul Bates* | 0.094 | - | 0.157 | - | - | - | - | - |
| Haruki Nakamura* | 0.141 | - | 0.223 | - | 0.079 | - | 0.092 | - |
| Sergei Grudinin* | 0.077 | - | 0.089 | - | 0.041 | - | 0.174 | - |
| SurFit, Daron Standley* | 0.066 | - | 0.084 | - | 0.129 | - | 0.248 | - |
| Xiaoqin Zou* | 0.163 | - | 0.247 | - | 0.044 | - | 0.054 | - |
| Zhiping Weng* | 0.224 | - | 0.217 | - | 0.214 | - | 0.310 | - |
| Jeffrey J. Gray* | 0.166 | - | 0.188 | - | - | - | - | - |
| Dmitry Korkin* | 0.039 | - | 0.012 | - | 0.077 | - | 0.070 | - |
| Juan Fernandez-Recio* | 0.295 | - | 0.278 | - | 0.172 | - | 0.289 | - |
| Yves Dehouck* | 0.286 | - | 0.252 | - | - | - | - | - |
| Huan-Xiang Zhou* | 0.165 | - | 0.236 | - | 0.147 | - | 0.161 | - |
| Mayuko Takeda-Shitaka* | 0.123 | - | 0.152 | - | 0.054 | - | 0.126 | - |
| Daisuke Kihara* | 0.054 | - | 0.179 | - | 0.015 | - | 0.098 | - |
| Yi Xiao* | 0.131 | - | 0.111 | - | - | - | - | - |
| Meng Cui* | 0.068 | - | 0.166 | - | - | - | - | - |
| Alexandre M.J.J. Bonvin* | - | - | - | - | 0.016 | - | 0.124 | - |

*These results are obtained directly from the CAPRI round 26 (7,8).

# Table S7. Ablation Study.

|  | S645 non-redundant | | SPIKE-ACE2 | | MDM2-p53 | | T55 | T56 |
| --- | --- | --- | --- | --- | --- | --- | --- | --- |
|  | Pearson | RMSE | Pearson | RMSE | Pearson | RMSE | Kendall | Kendall |
| Full architecture | 0.34 | 1.95 | 0.37 | 1.43 | 0.37 | 0.64 | 0.20 | 0.27 |
| without gbs2D  (Conv + TransEncoder) | 0.26 | 2.03 | 0.39 | 1.39 | 0.25 | 0.88 | 0.23 | 0.22 |
| without others1D  (Dense1 + Dense 2) | 0.30 | 1.96 | 0.18 | 1.53 | 0.24 | 0.59 | 0.21 | 0.14 |
| without GCN | 0.29 | 2.00 | 0.40 | 1.44 | 0.41 | 0.87 | 0.26 | 0.24 |

# Table S8. Feature importance.

| Shuffled features | S645 non-redundant testing | |
| --- | --- | --- |
|  | Pearson | RMSE |
| None | 0.343 | 1.953 |
| All | 0.269 | 2.018 |
| FoldX | 0.300 | 1.986 |
| Δ *authority score* | 0.330 | 1.963 |
| Others | 0.336 | 1.973 |

# Table S9. Performance comparison on multiple mutation datasets.

|  | SM1124 | | | SM595 | | | SM_ZEMu | | |
| --- | --- | --- | --- | --- | --- | --- | --- | --- | --- |
| Methods/Group | *R*^2^ | Pearson | RMSE | *R*^2^ | Pearson | RMSE | *R*^2^ | Pearson | RMSE |
| DDMut-PPI | 0.65 | 0.83 | 1.51 | 0.18 | 0.71 | 2.56 | 0.33 | 0.72 | 2.04 |
| mmCSM-PPI | 0.61 | 0.78 | 1.58 | 0.47 | 0.69 | 2.06 | 0.52 | 0.73 | 1.72 |
| FoldX | -0.51 | 0.40 | 3.12 | -5.85 | 0.08 | 7.39 | -1.38 | 0.53 | 3.83 |
| Discovery Studio | - | - | - | -0.18 | 0.39 | 3.07 | - | - | - |
| ZEMu | - | - | - | - | - | - | 0.28 | 0.64 | 2.11 |

# Table S10. Performance comparison on SM1124 subsets increasing and decreasing affinity.

|  | Increasing affinity (n = 204) | | | Decreasing affinity (n = 900) | | |
| --- | --- | --- | --- | --- | --- | --- |
| Methods/Group | *R*^2^ | Pearson | RMSE | *R*^2^ | Pearson | RMSE |
| DDMut-PPI | -0.81 | 0.65 | 1.65 | 0.54 | 0.81 | 1.48 |
| mmCSM-PPI | -0.99 | 0.33 | 1.73 | 0.50 | 0.73 | 1.55 |
| FoldX | -5.42 | 0.15 | 3.10 | -1.04 | 0.29 | 3.13 |


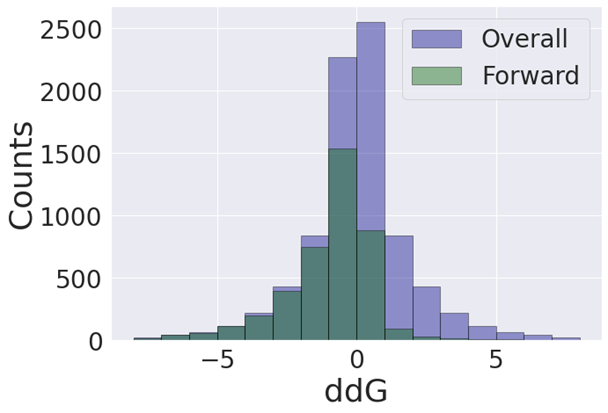


# Figure S1. ΔΔG distribution of single point mutation training set. The distribution of the original dataset S4169 is coloured green (Forward). The distribution of S8338 after adding the hypothetical reverse mutations is coloured purple (Overall).


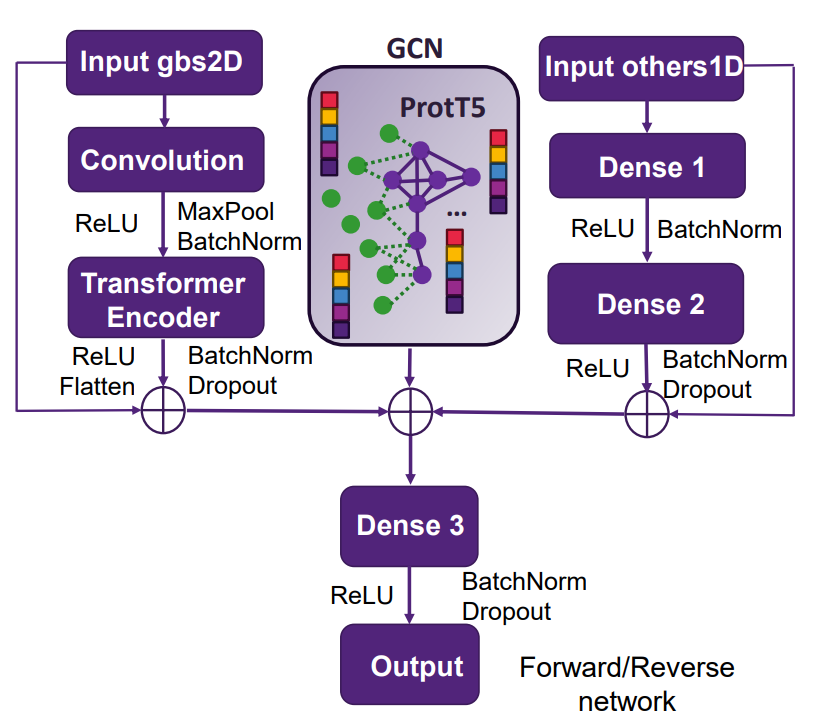


# Figure S2. Model architecture for each sub-component in the siamese network. Graph-based signatures are processed by a Convolutional layer and Transformer Encoder, other tabular features are processed by two Dense layers, and the interface graph are processed by a Graph Convolutional Network. Then they are concatenated and output the predictions.

# Manual S1. Running predictions for single point mutations.

## Part A. User Input Mutations


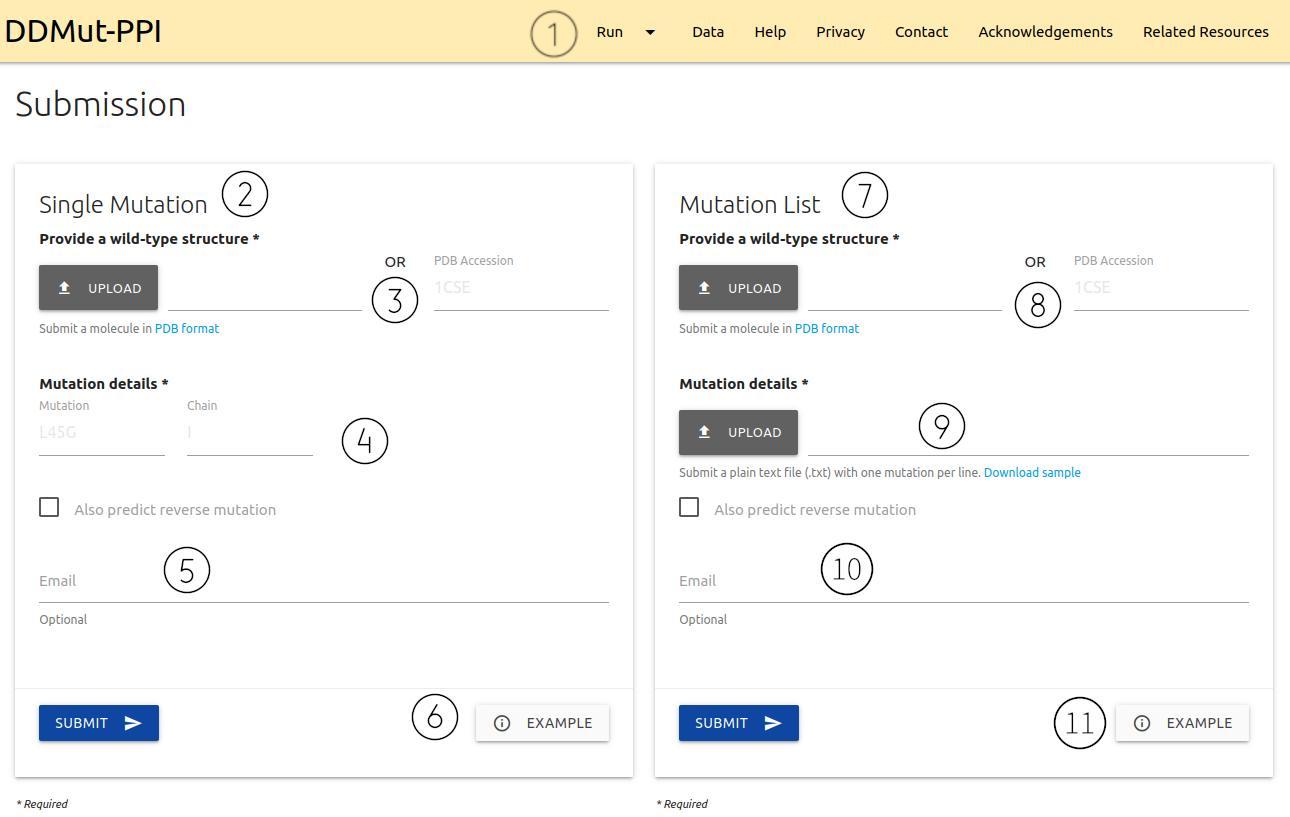


The submission page for user input mutations can be accessed via the menu item Run (1) on the top bar menu.

- The *Single Mutation* (2) option allows the user to submit one single mutation for prediction.
- Users are required to provide a wild-type structure (3) by uploading a file in PDB format or providing a PDB accession code.
- Details on the single mutation should be provided (4) A mutation code consists of a *wild-type* code, *residue* position and *mutant* code (using the one letter amino acid code). The residue position must be consistent with the PDB file. The *chain* for the mutation must also be provided.
- If an email address is provided (5), a link to the results page will be sent to the user as soon as DDMut-PPI finishes processing the submission.
- An example of the results page is also provided (6).
- The *Mutation List* (7) option allows the user to submit a list of single mutations to be analysed by DDMut-PPI in batch.
- Similarly to the *Single Mutation* option, a wild-type structure is required (8) and can be input by uploading a file in PDB format or providing a PDB accession code.
- The list of single-point mutations must be input (9) by uploading a file containing the mutation codes (*chain* identifier, *wild-type* residue code, *position,* and *mutant* residue code). The file format requires one mutation per line. A sample file is also provided.
- If an email address is provided (10), a link to the results page will be sent to the users soon as DDMut-PPI finishes processing the submission.
- An example of the results page is also provided (11).

## Part B. Interface Analysis


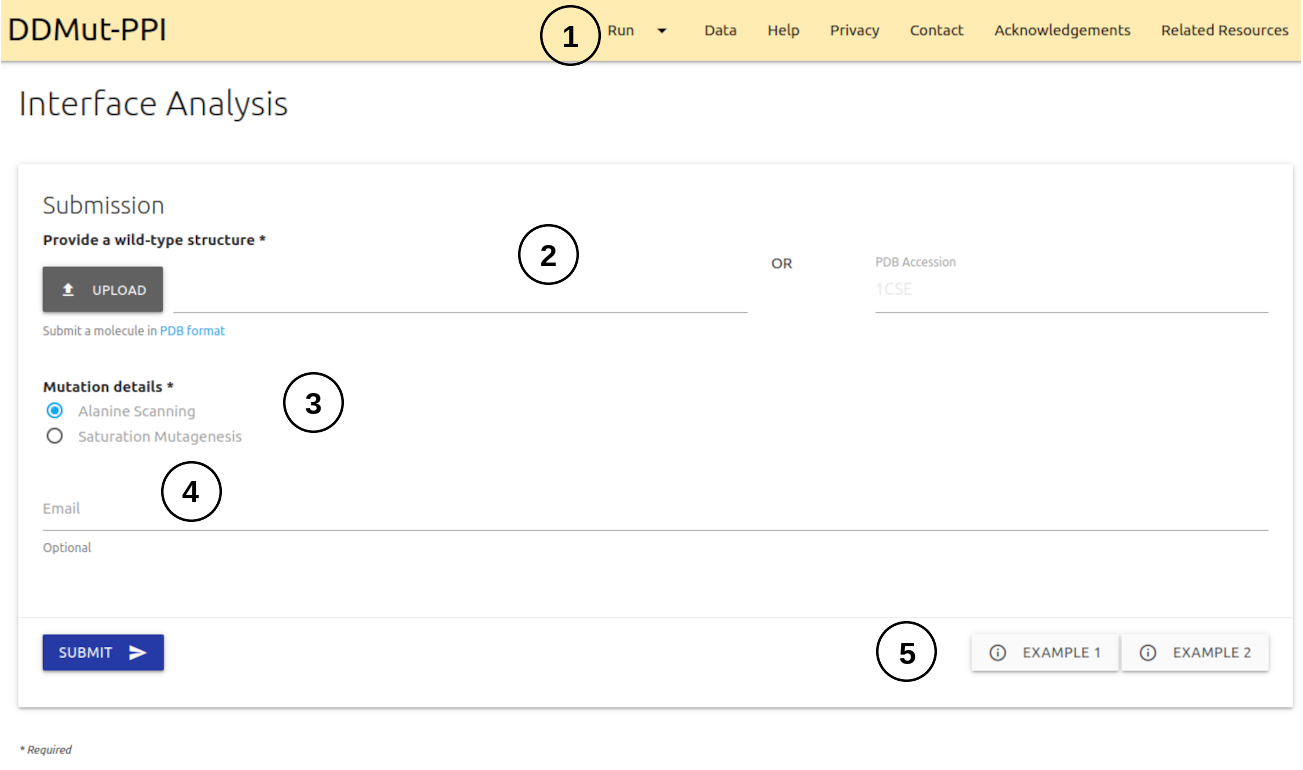


The submission page for automated interface analysis can be accessed via the menu item Run (1) on the top bar menu.

- Users are required to provide a wild-type structure (2) by uploading a file in PDB format or providing a PDB accession code.
- On *Mutation Details* section (3), one is required to provided which type of analysis to run. Here two options are available: Alanine Scanning (mutate all residues at the interface to Alanine) or Saturation Mutagenesis (mutate all residues at the interface to all nineteen standard amino acids).
- If an email address is provided (4), a link to the results page will be sent to the user as soon as DDMut-PPI finishes processing the submission.
- An example of the results page is also provided (5).

# Manual S2. Running predictions for multiple point mutations.


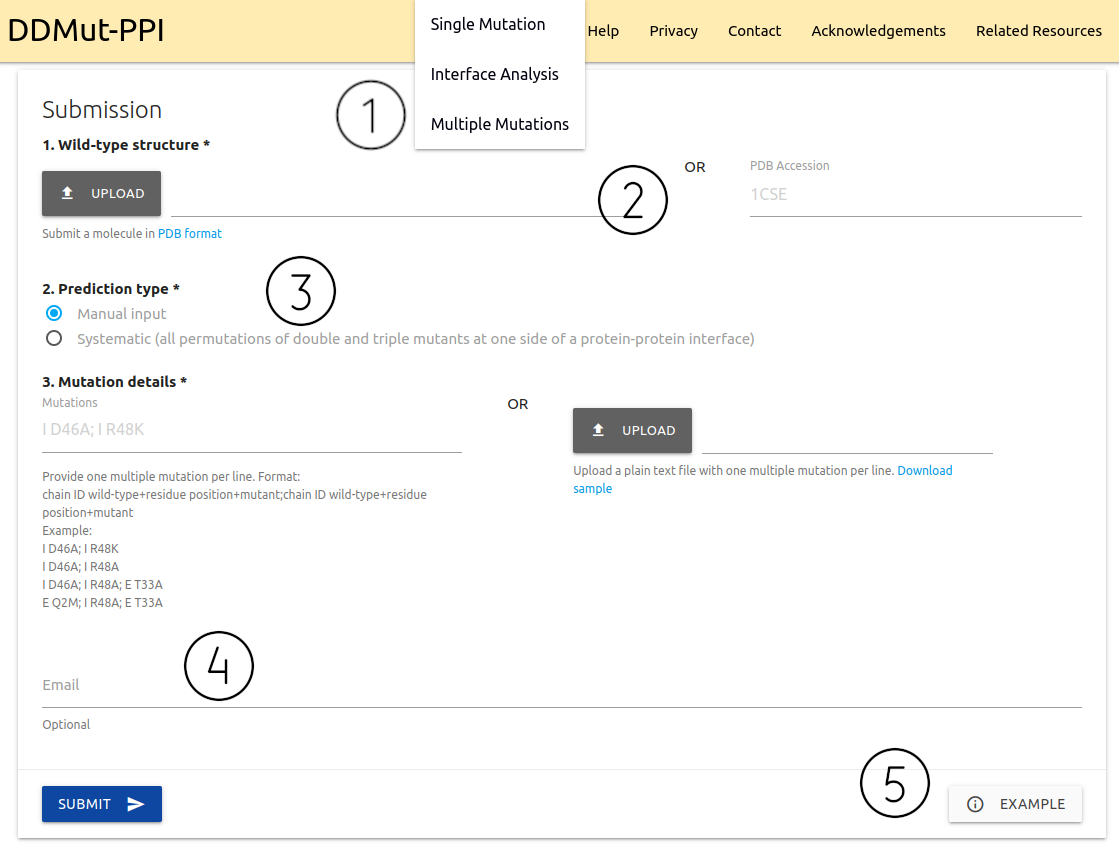


The input page can be accessed from the top menu Run (1).

Users are required to provide a protein structure by uploading a file in [PDB format](https://www.wwpdb.org/documentation/file-format) or typing a PDB four letter accession code (2).

Two prediction types are available (3):

- Manual: This option allows for the input of specific multiple mutations which can be provided via the text field or by uploading a plain text file with a list of entries. In both cases, multiple mutations must be separated by a semi-colon (;) and one entry per line. Input examples and a [sample file](https://biosig.lab.uq.edu.au/ddmut_ppi/static/mutations.txt) are available.
- Systematic: If this option is selected, users are required to provide a chain identifier from which interfaces residues will be identified and all permutations of double and triple mutants generated for evaluation. The results page will show the top one hundred entries increasing affinity and top one hundred decreasing affinity.

If provided (4), an email will be sent to the user after the submission is processed.

A button for an example results page is available at the bottom of the form (5)

# Manual S3. Results for single point mutations.

## Part A. Single Mutation


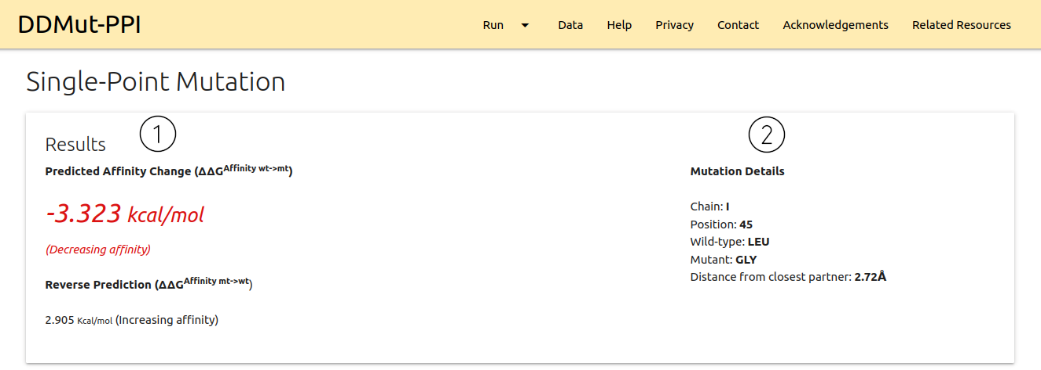

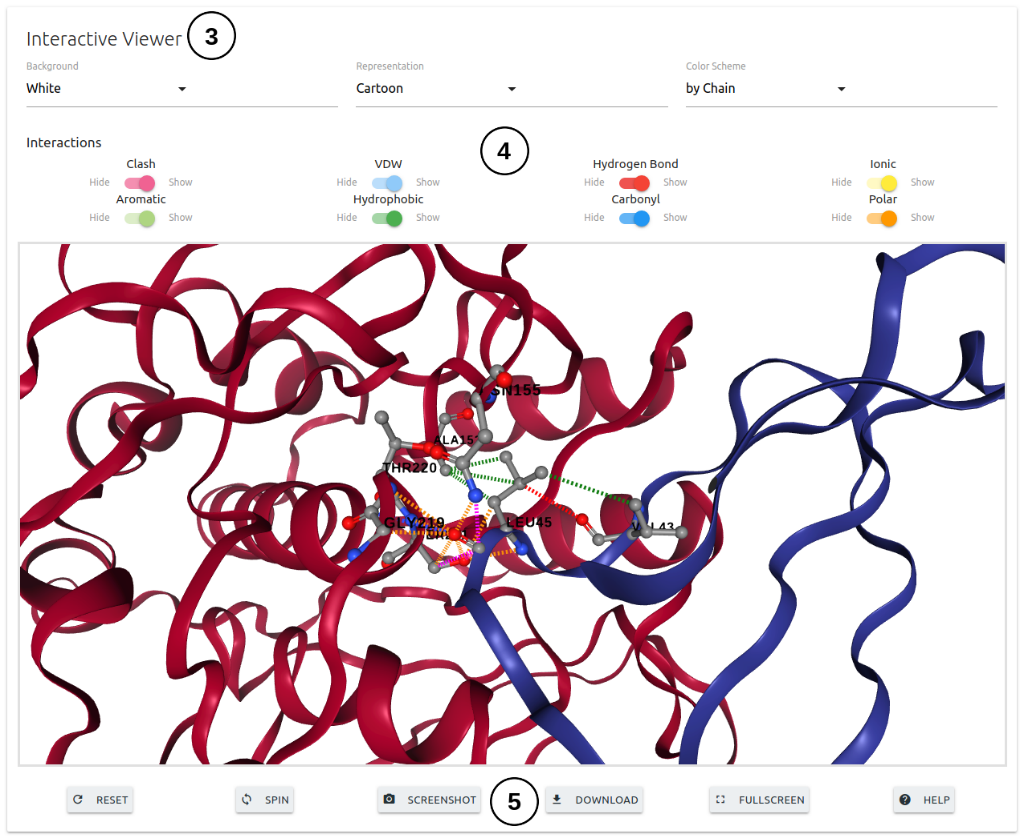

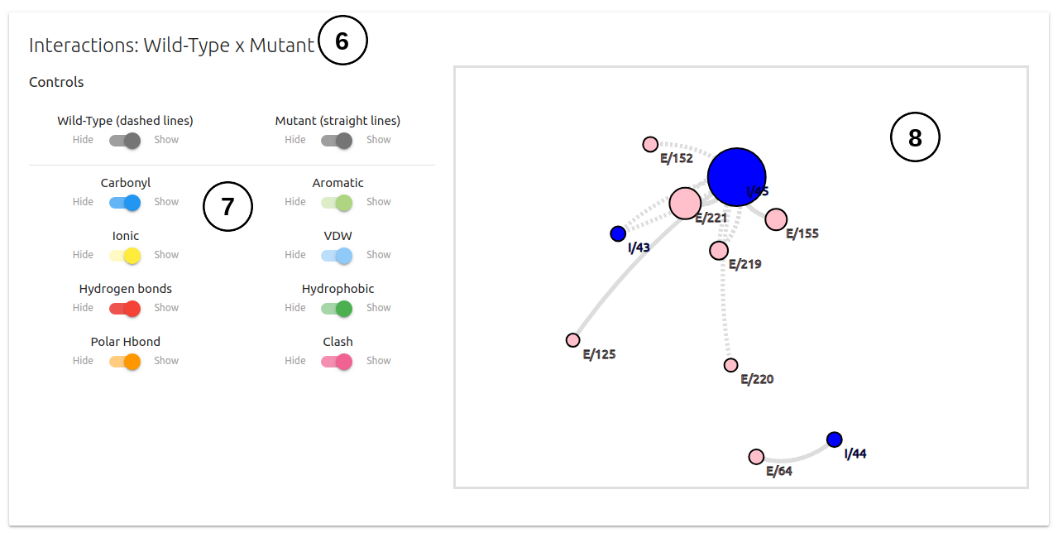


The results page for a single mutation will be shown once computations are completed on three different panels.

- On the first panel, the ΔΔG is displayed (1) alongside with the details on the mutation being analysed (2).
- The second panel will show an interactive 3D viewer (3), which allows user to analyse the interatomic interactions between the wild-type residue and nearby residues. Interactions can be hidden or displayed using the controls provided (4). The viewer can also be manipulated using buttons at the bottom of the panel (5).
- Lastly, on the third panel, DDMut-PPI allows users to analyse the inter-residue interactions of wild-type and mutant (6). Here, a set of switches are also provided (7) to allow customization of the interactions being displayed on the side box (8).

## Part B. Mutation List


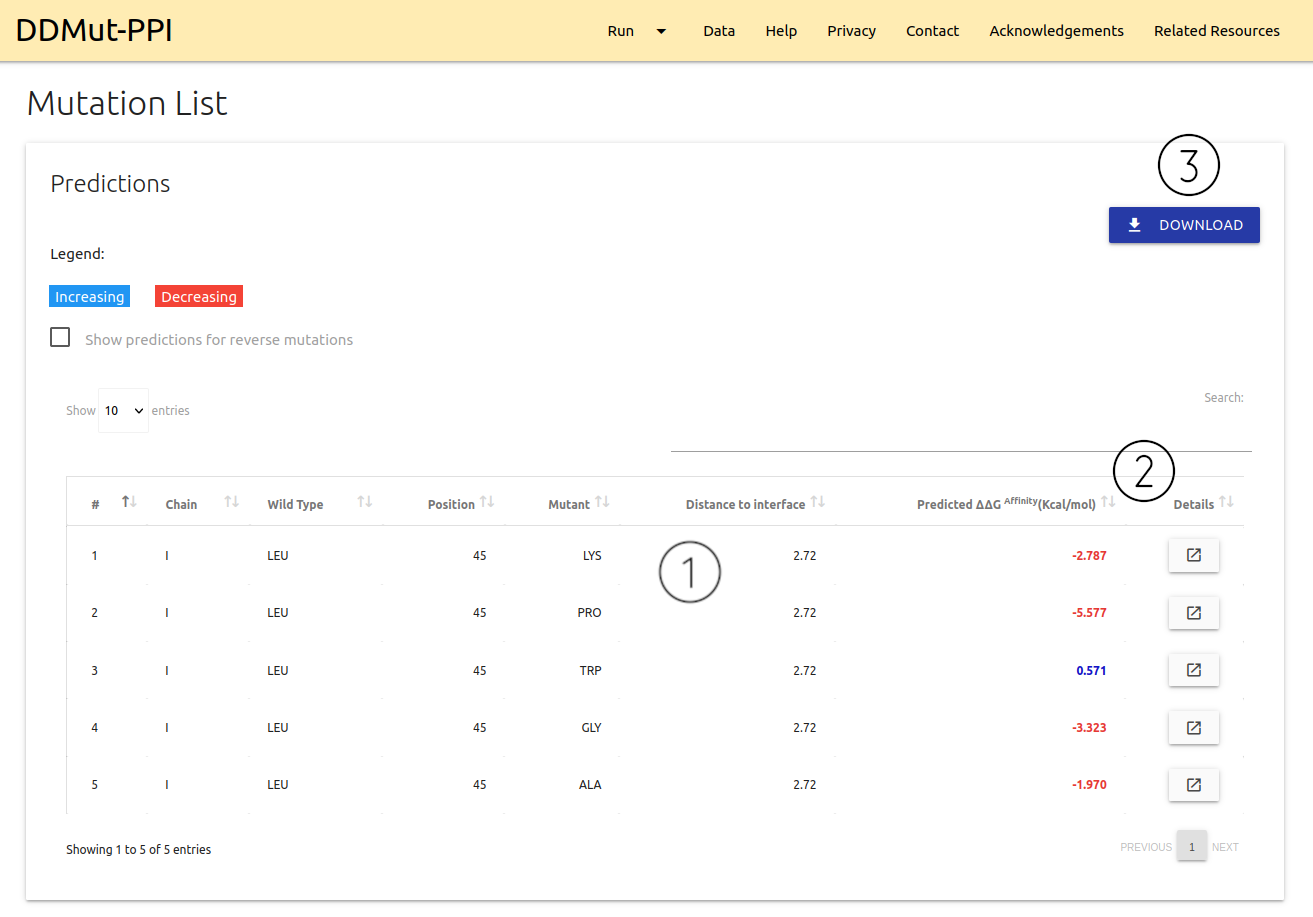


The results page for a list of single mutations will be shown once computations are completed in a single table.

- The predicted ΔΔG for every single-point mutation will be displayed on a table (1). A set of controls for sorting based on columns and an input for text search are also provided.
- All the analysis discussed for the *Single Mutation* option can be analysed for each single mutation on the table through the Details button (2) of each row.
- Results can be downloaded as a .csv file by clicking on the Download button on the top right corner (3).

## Part C. Interface Analysis


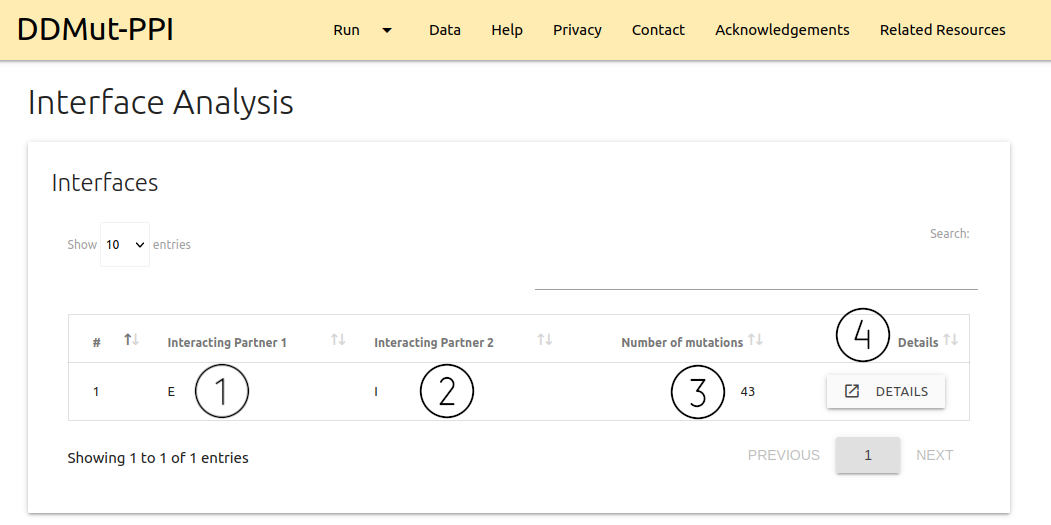


## Part D. Alanine Scanning


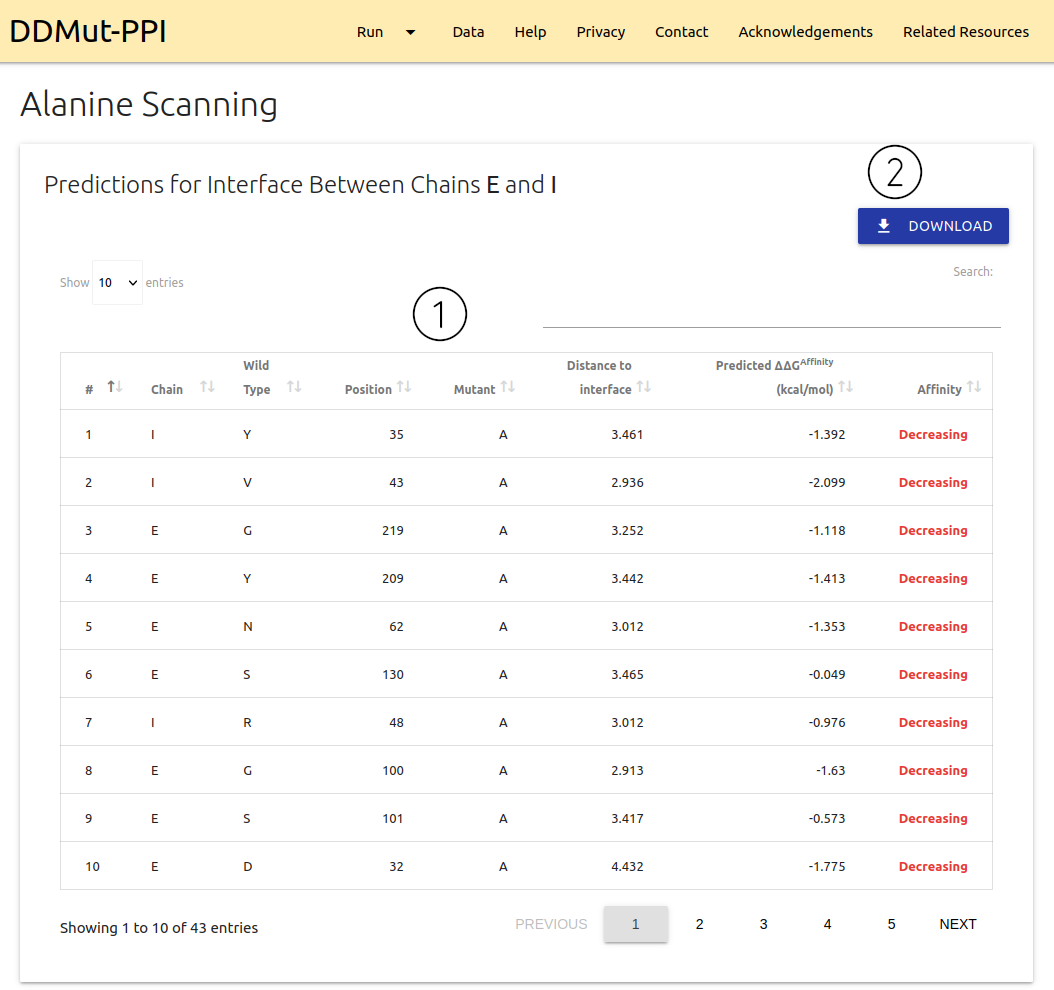


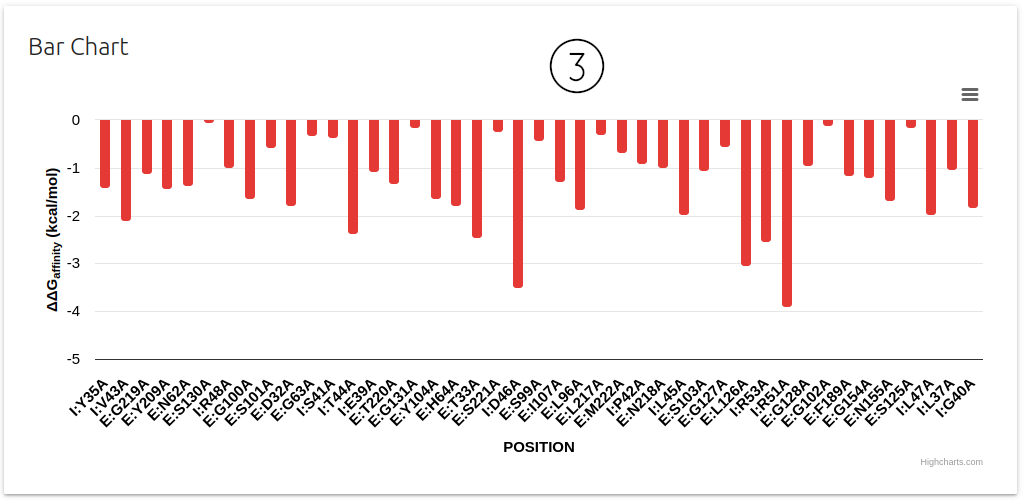

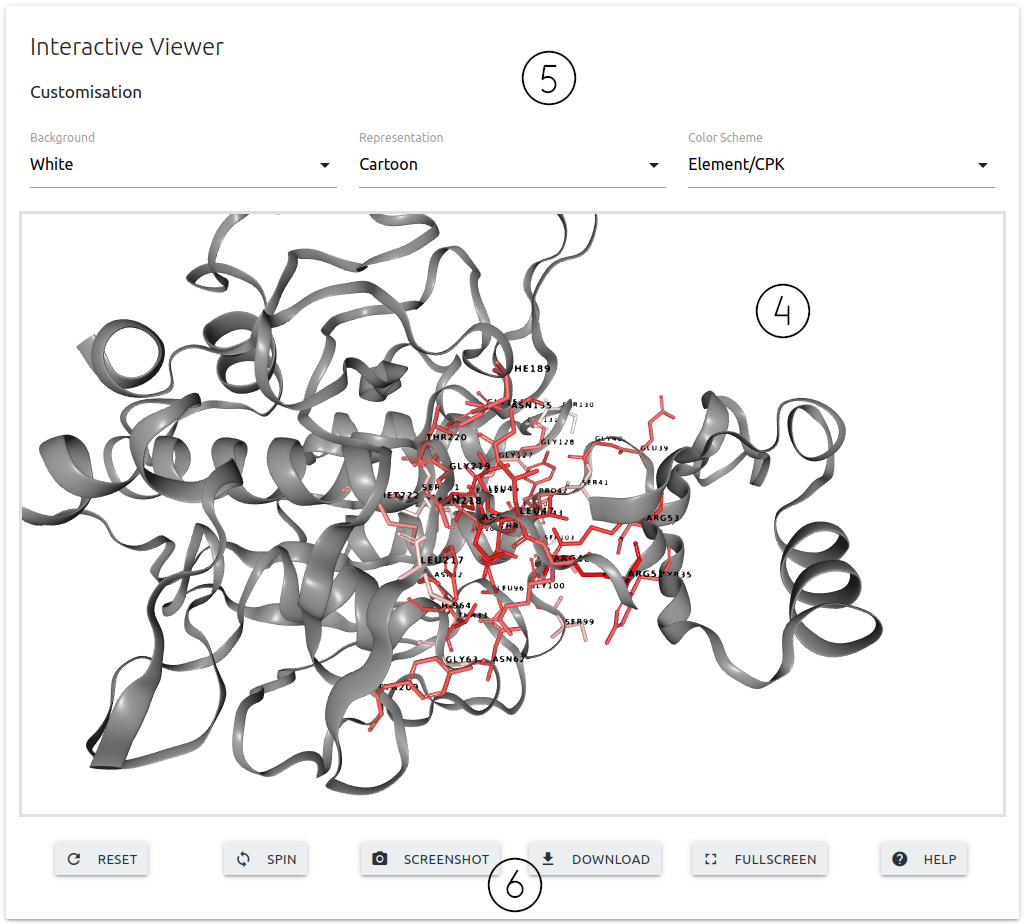


The results page for Alanine Scanning will be displayed on three different panels.

- The predicted ΔΔG for every single-point mutation to Alanine will be displayed on a table (1) on the first panel. A set of controls for sorting based on columns and an input for text search are also provided.
- Results can be downloaded as a .csv file by clicking on the Download button on the top right corner (2).
- All results are also summarized on a bar plot on the second panel (3). Positive and negative ΔΔG predictions are shown in in blue and red, respectively.
- The third panel will show and interactive 3D viewer (4) in which the interface residues are coloured according to the ΔΔG predictions. The viewer can be customized using the controls on the top of the panel (5). A set of buttons are displayed at the bottom of the panel (6) to adjust visualization.

## Part E. Saturation Mutagenesis


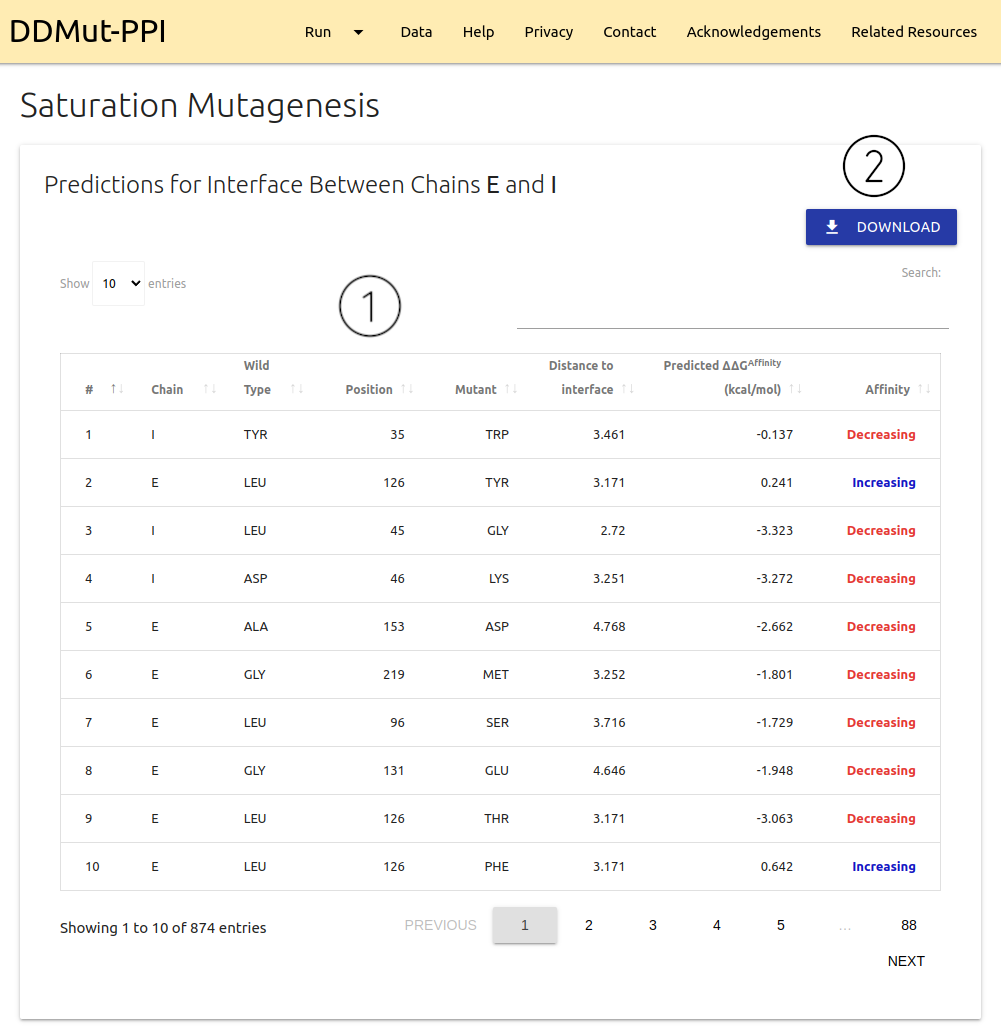


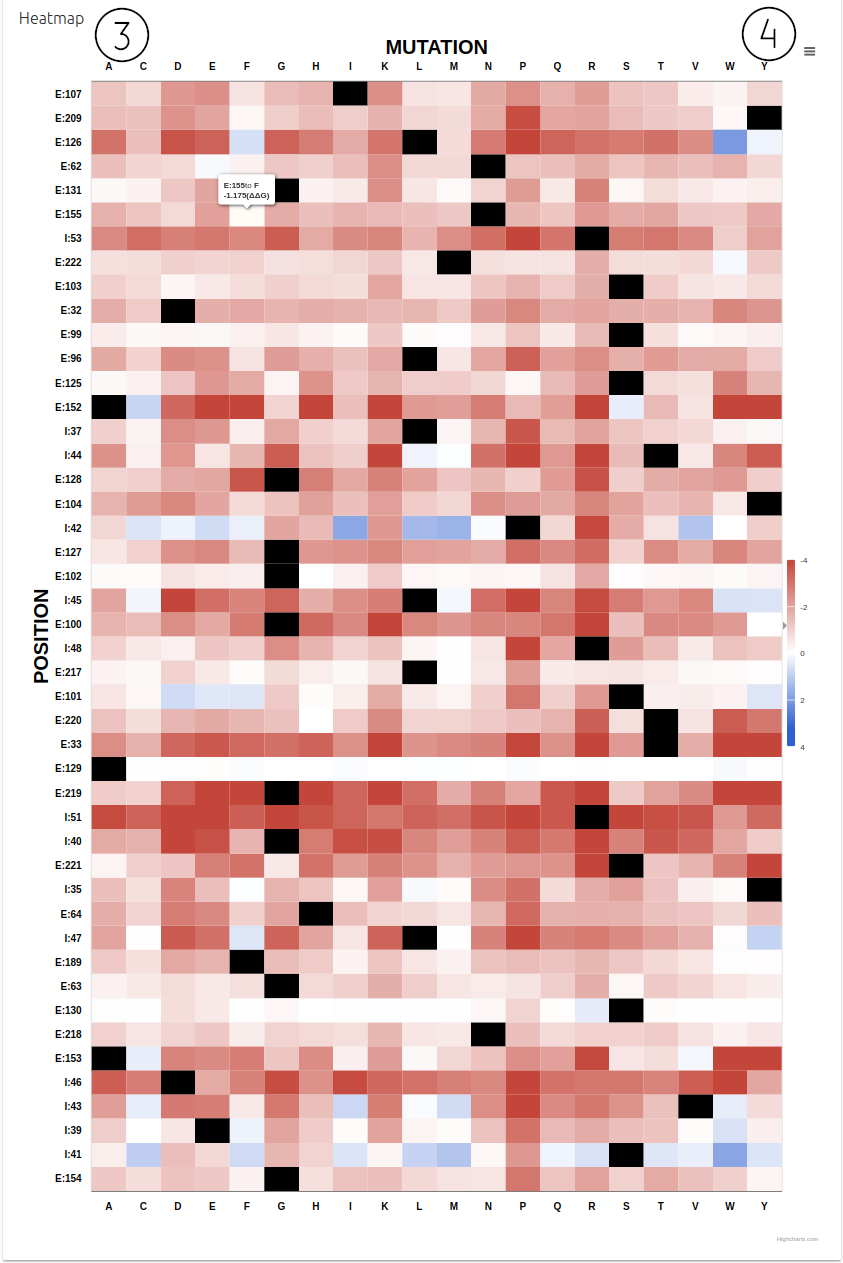

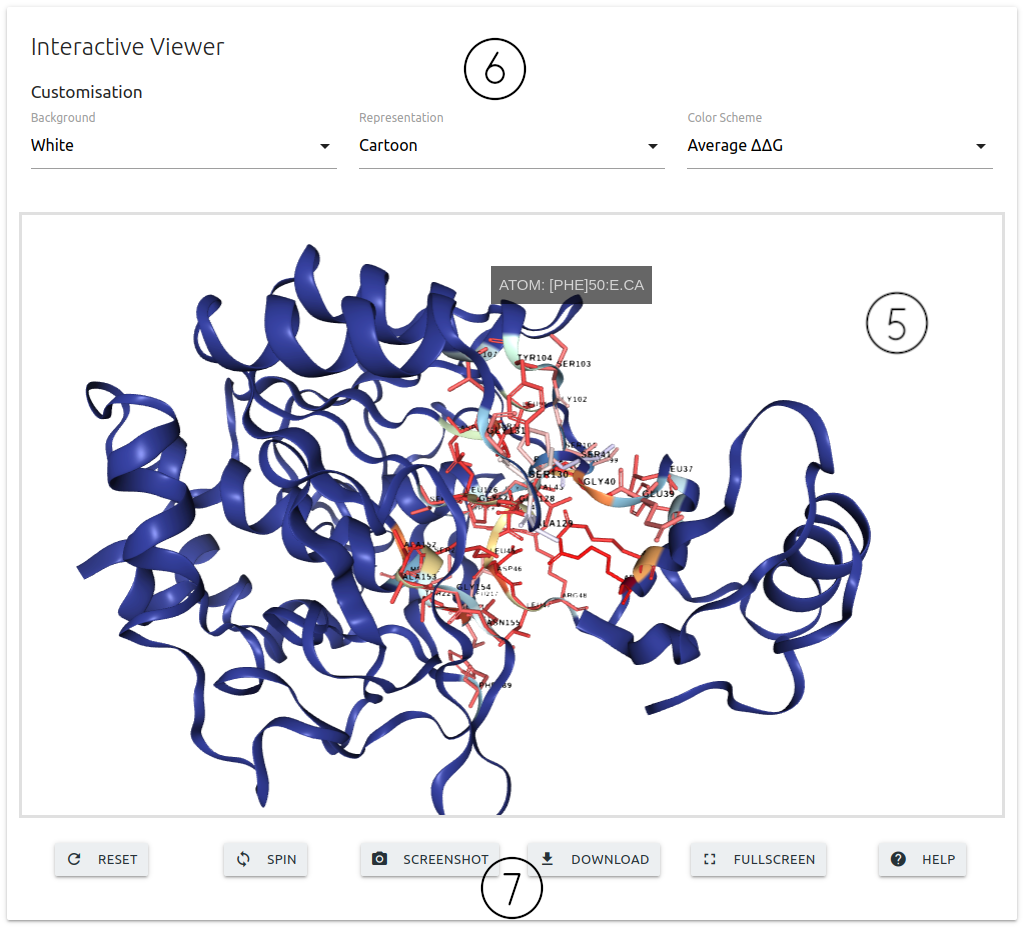


The results page for Saturation Mutagenesis will also be displayed on three different panels.

- The predicted ΔΔG for every single-point mutation to Alanine will be displayed on a table (1) on the first panel. A set of controls for sorting based on columns and an input for text search are also provided.
- Results can be downloaded as a .csv file by clicking on the Download button on the top right corner (2)
- All results are also summarized on a heatmap plot on the second panel (3) which can be downloaded as an image file (4).
- The third panel will show and interactive 3D viewer (5) in which the interface residues are coloured according to the average ΔΔG predictions values. The viewer can be customized using the controls on the top of the panel (6). A set of buttons are displayed at the bottom of the panel (7) to adjust visualization.

# Manual S4. Results for multiple point mutations.


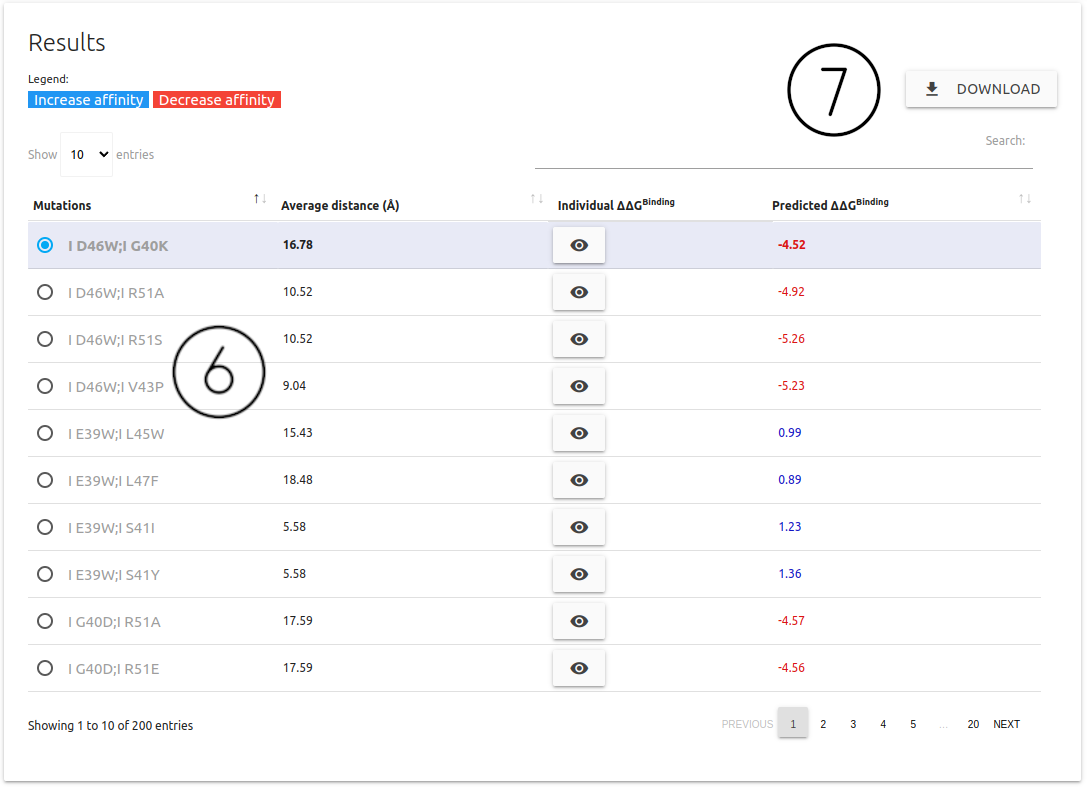


- For both types of prediction (manual and systematic), results are shown in table format (6) where entries predicted as increasing affinity are shown in blue and decreasing affinity in red. Individual ΔΔG^Binding^ for each point mutation can also be viewed by clicking the button visibility. These are calculated using DDMut-PPI single mutation model.
- A button for downloading (7) all the results as a comma separated file (csv) is available.
- Systematic evaluations of PPI interface show the top one hundred increasing/decreasing affinity entries based on all permutations of double and triple mutants at one side of a protein-protein interface.


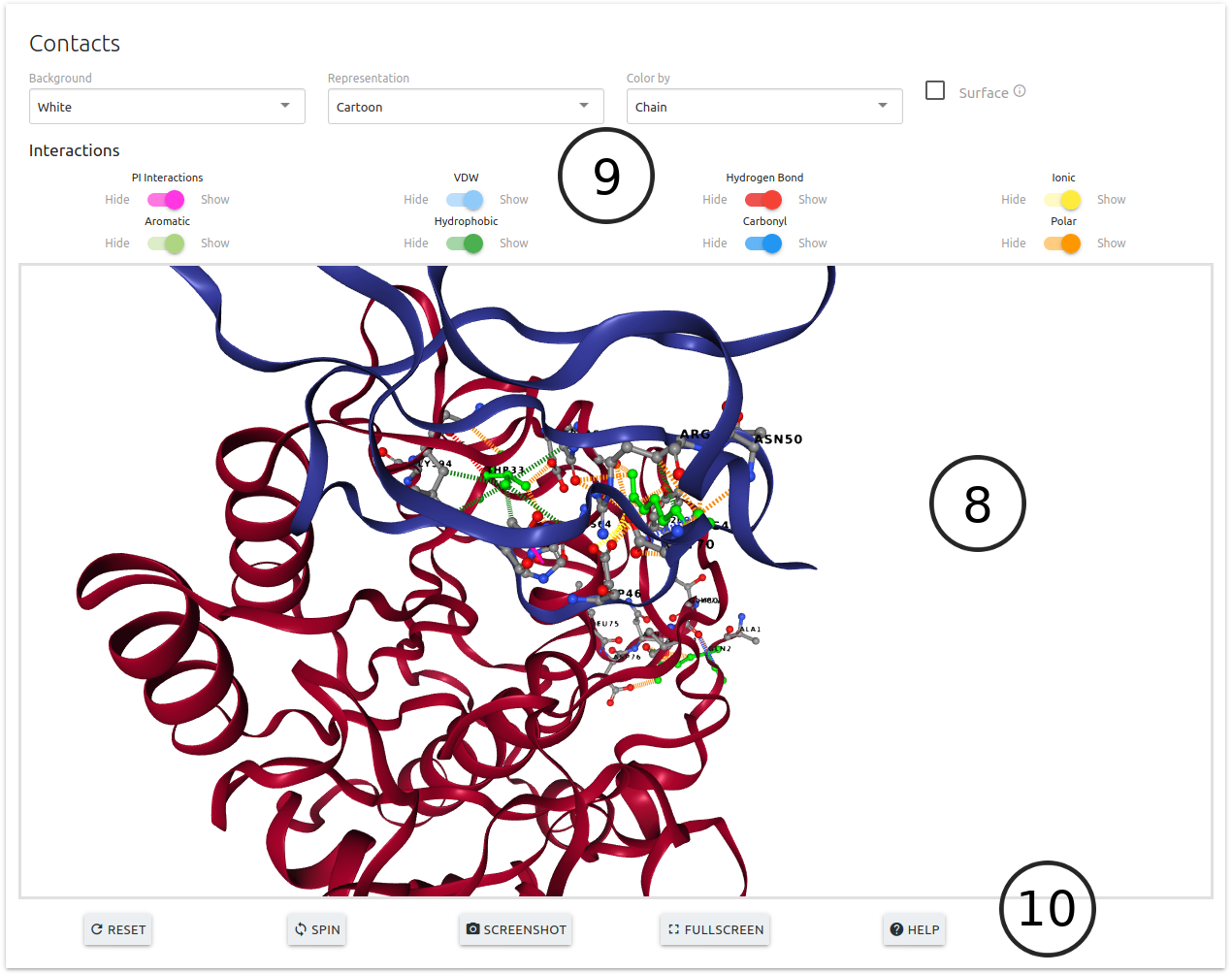


- An interactive 3D viewer is available (8) allowing for the analysis of interatomic interactions for the wild-type residues. A set of controls are also available for customizing the viewer (9)
- Action buttons at the bottom of the viewer (10)

# References

1. Jiang, Y., Quan, L., Li, K., Li, Y., Zhou, Y., Wu, T. and Lyu, Q. (2023) DGCddG: Deep Graph Convolution for Predicting Protein-Protein Binding Affinity Changes Upon Mutations. *IEEE/ACM Trans Comput Biol Bioinform*, 20, 2089-2100.

<http://www.ncbi.nlm.nih.gov/pubmed/37018301>

<http://dx.doi.org/10.1109/TCBB.2022.3233627>

2. Rodrigues, C.H.M., Myung, Y., Pires, D.E.V. and Ascher, D.B. (2019) mCSM-PPI2: predicting the effects of mutations on protein-protein interactions. *Nucleic Acids Res*, 47, W338-W344.

<http://www.ncbi.nlm.nih.gov/pubmed/31114883>

<http://dx.doi.org/10.1093/nar/gkz383>

3. Li, M., Simonetti, F.L., Goncearenco, A. and Panchenko, A.R. (2016) MutaBind estimates and interprets the effects of sequence variants on protein-protein interactions. *Nucleic Acids Res*, 44, W494-501.

<http://www.ncbi.nlm.nih.gov/pubmed/27150810>

<http://dx.doi.org/10.1093/nar/gkw374>

4. Liu, Y., Armstrong, G., Tam, J. and Chen, B.Y. (2023) MechPPI: Binding Mechanism-based Machine-Learning tool for Predicting Protein-Protein Binding Affinity Changes Upon Mutations. *bioRxiv*, 2023.2010.2026.564257.

<http://dx.doi.org/10.1101/2023.10.26.564257>

5. Pires, D.E. and Ascher, D.B. (2016) mCSM-AB: a web server for predicting antibody-antigen affinity changes upon mutation with graph-based signatures. *Nucleic Acids Res*, 44, W469-473.

<http://www.ncbi.nlm.nih.gov/pubmed/27216816>

<http://dx.doi.org/10.1093/nar/gkw458>

6. Wang, M., Cang, Z. and Wei, G.W. (2020) A topology-based network tree for the prediction of protein-protein binding affinity changes following mutation. *Nat Mach Intell*, 2, 116-123.

<http://www.ncbi.nlm.nih.gov/pubmed/34170981>

<http://dx.doi.org/10.1038/s42256-020-0149-6>

<http://www.ncbi.nlm.nih.gov/pmc/articles/Competing> interestsThe authors declare no competing interests.

7. Whitehead, T.A., Chevalier, A., Song, Y., Dreyfus, C., Fleishman, S.J., De Mattos, C., Myers, C.A., Kamisetty, H., Blair, P., Wilson, I.A. and Baker, D. (2012) Optimization of affinity, specificity and function of designed influenza inhibitors using deep sequencing. *Nat Biotechnol*, 30, 543-548.

<http://www.ncbi.nlm.nih.gov/pubmed/22634563>

<http://dx.doi.org/10.1038/nbt.2214>

<http://www.ncbi.nlm.nih.gov/pmc/articles/COMPETING> FINANCIAL INTERESTS The authors declare competing financial interests: details are available in the online version of the paper.

8. Janin, J., Henrick, K., Moult, J., Eyck, L.T., Sternberg, M.J., Vajda, S., Vakser, I., Wodak, S.J. and Critical Assessment of, P.I. (2003) CAPRI: a Critical Assessment of PRedicted Interactions. *Proteins*, 52, 2-9.

<http://www.ncbi.nlm.nih.gov/pubmed/12784359>

<http://dx.doi.org/10.1002/prot.10381>
